# Prognostic and Clinicopathological Correlations of Cell Cycle Marker Expressions before and after the Primary Systemic Therapy of Breast Cancer

**DOI:** 10.1007/s12253-019-00726-w

**Published:** 2019-08-24

**Authors:** Tímea Tőkés, Anna-Mária Tőkés, Gyöngyvér Szentmártoni, Gergő Kiszner, Dorottya Mühl, Béla Ákos Molnár, Janina Kulka, Tibor Krenács, Magdolna Dank

**Affiliations:** 1 Oncology Center, Semmelweis University, Tömő utca 25-29, 4th floor, Budapest, H-1083 Hungary; 2 2nd Department of Pathology, Semmelweis University, Üllői út 93, Budapest, H-1091 Hungary; 3 1st Department of Pathology and Experimental Cancer Research, Semmelweis University, Üllői út 26, Budapest, H-1085 Hungary; 4 1st Department of Surgery, Semmelweis University, Üllői út 78/A, Budapest, H-1083 Hungary

**Keywords:** Breast cancer, Primary systemic therapy, Cell cycle, Ki67, MCM, Cyclin A, PHH3

## Abstract

We aimed to analyze the expression of cell-cycle regulation markers – minichromosome maintenance protein 2 (MCM2), Ki-67, Cyclin-A and phosphohistone-H3 (PHH3) − in pre-treatment core-biopsy samples of breast carcinomas in correlation with known predictive and prognostic factors. Totally 52 core biopsy samples obtained prior to neoadjuvant therapy were analyzed. Immunohistochemistry was performed to analyze the expression of MCM2, Ki-67, Cyclin A and PHH3, which were correlated with the following clinicopathological parameters: clinical TNM, tumor grade, biological subtype, the presence of tumor infiltrating lymphocytes (TIL), pathological tumor response rate to the neoadjuvant therapy and patient survival. All investigated markers showed higher expression in high grade and in triple negative tumors (*p* < 0.01 and *p* < 0.05, respectively). Hormone receptor negative tumors showed significantly higher expression of Ki-67 (p < 0.01), MCM2 (p < 0.01) and Cyclin A (p < 0.01) than hormone receptor positive ones. Tumors with increased TIL showed significantly higher Ki-67 expression (*p* = 0.04). Pattern analysis suggested that novel cell-cycle marker-based subgrouping reveals predictive and prognostic potential. Tumors with high MCM2, Cyclin A or PHH3 expression showed significantly higher rate of pathological complete remission. Tumors with early relapse (progression-free survival ≤2 years) and shortened overall survival also show a higher rate of proliferation. Our cell cycle marker (Ki-67, MCM2, Cyclin A, PHH3) based testing could identify tumors with worse prognosis, but with a favorable response to primary systemic therapy. The pattern of cell-cycle activity could also be useful for predicting early relapse, but our findings need to be further substantiated in larger patient cohorts.

**Electronic supplementary material:**

The online version of this article (10.1007/s12253-019-00726-w) contains supplementary material, which is available to authorized users.

## Background

In breast carcinomas several predictive and prognostic markers have already been defined and used in the daily clinical routine, i.e. TNM stage, tumor grading and biological subtyping [1, 2]. In locally advanced breast cancers, primary systemic therapy (PST) is the recommended first therapeutic approach [1, 3]. However, there is still a need to find reliable and reproducible biomarkers, which can predict pathologic complete remission (pCR) and patients’ prognosis, in order to select those who surely benefit from PST. The markers of cell proliferation are promising candidates for this clinical goal [4]. Ki-67 labeling index (Ki-67 LI) is routinely used to assess proliferation activity of breast tumors [1, 2]. However, it should be highlighted that contradictory data are presented about the reliability of Ki-67 LI to assess tumor proliferation due to its relatively low inter-laboratory reproducibility [5, 6].

In case of breast carcinoma only a few studies are available which are investigating the predictive and prognostic role of cell cycle markers besides the Ki-67 LI [7–10]. The minichromosome maintenance protein complex (MCM2–7), Cyclin A, E and D1, geminins, Aurora kinase A and B and the phosphohistone-H3 (H3S10ph ~ referred to as PHH3) are the most frequently investigated biomarkers in these studies [4, 7]. Our research group aimed to test the role of MCM2, Cyclin A and PHH3 as potential biomarkers predicting response to treatment and clinical outcome of locally advanced breast cancer.

Like Ki-67, the MCM-complex is expressed during the whole cell cycle, except for G0 [11]. The MCM complex contains six replicative helicases (MCM2–7) which play a crucial role in replication initiation/licensing. Expression of these proteins cover a wider range of cycle than does Ki-67 of which the function starts in G1-S, and stays active until the anaphase, connecting to the condensing chromosomes in G2 [11]. MCMs are already expressed in cells committed toward cell-division and are part of the pre-replication complex, as well [12, 13]. Loddo et al. also highlighted the importance of S/G2 and M-phase markers besides MCM2, to form further cell-cycle activity-based subgroups of breast cancer with different prognostic potential [7]. For the former, Cyclin A is a good example, as being an S-phase checkpoint and DNA-repair regulator, expressed from the S-phase till its role in the G2/M-transition. Therefore it is a suitable marker of the S/G2 phases [7, 14]. As an independent M-phase marker, we used PHH3 in our current study. PHH3 is one of the proteins responsible for chromatin condensation; therefore PHH3 is an M-phase marker accurately reflecting the mitotic activity of the cell [15–17].

Pre-therapy Ki-67 LI (measured in core-biopsies before the initiation of PST) have already been proved to be predictive for pCR in breast cancer patients, but the best predictive cut-off point for the favorable outcome is under debate [18–20]. In our earlier published study [21] we proved that – besides the Ki-67 LI – the higher expression of MCM2, Cyclin A and PHH3 also accurately anticipates pCR. For these cell-cycle markers we also defined predictive cut-off points to pCR for the clinical routine, to improve objectivity.

Besides its predictive value the prognostic potential of Ki-67 – namely that high expression of Ki-67 is associated with a poor prognosis and with an earlier onset of metastatic disease – has also been confirmed, but different prognostic cut-off points were defined in the earlier published literature [18–20]. It was also higlighted by Fasching et al., that the predictive and the prognostic cut-off points must be defined separately for the Ki-67 LI. In their study they defined 40% positivity cut-off point for pCR alone and a 13% positivity cut-off point to be prognostic for both pCR and OS [22]. The 13th St. Gallen consensus defined a 14% positivity cut-off point to differentiate prognostic groups of breast cancer based on the biological behaviour of the tumors, but later suggested a different, namely 20% cut-off to be used for response prediction [2]. In our earlier published study [23] we defined a 20% prognostic cut-off of Ki-67 LI for progression-free survival (PFS) and a 30% for overall survival (OS), respectively, but as a predictive cut-off, 45% was specified [21].

In addition to Ki-67, MCM2 expression also seems to have prognostic value in breast cancer [8, 12]. MCM2 positivity ratio of over 30% was found to be prognostic for poorer clinical outcome by Loddo et al. [7]. Cyclin A also has possible prognostic potential in breast cancers: higher Cyclin A expression (over 8.5%–10.5%) is associated with worse prognosis [24–26]. PHH3 based mitotic-index proved to be a stronger prognostic factor than the regularly applied mitotic activity index measured on hematoxylin-eosin stained slides [15–17]. According to Skaland et al., if the rate of M-phase cells was over 13 mitosis/10 NNL it was associated with poor prognosis [17].

In our current study we aimed to further investigate the correlation between these nuclear protein markers either expressed throughout (Ki67, MCM2), from post G1 (Cyclin A) or in M-phase (PHH3) of the cell cycle and the routinely examined clinicopathological factors of breast cancers undergoing PST (i.e. histological type, tumor grade, cTNM stage, biological behavior and tumor subtypes). Additionally, a novel prognostic biomarker, the presence of tumor infiltrating lymphocytes (TIL) was also assessed in our study. Over its prognostic significance, the predictive value of TIL infiltration is under debate in the neoadjuvant setting and its association with tumor proliferation has not been investigated thoroughly yet [27]. Prognostic value of the investigated cell cycle markers were also assessed regarding PFS and OS. For histological analyses and immunohistochemistry we used core-biopsy samples taken before the initiation of the neoadjuvant treatment.

## Materials and Methods

### Study Population

Patients diagnosed with primary breast cancer and treated with PST at the Oncology Center of Semmelweis University were retrospectively identified. Inclusion and exclusion criteria were the same as applied in our earlier study published previously [21]. Briefly, inclusion criteria were the following: (1) diagnosis of breast cancer (between 2008 and 2014) confirmed by ultrasound guided core biopsy sampling; (2) first oncological treatment was PST, initiated between 1 January 2008 and 31 December 2013 (end of follow up: 31 May 2017); (3) lack of any distant metastasis at the time of breast cancer diagnosis confirmed by ^18^F-fluorodeoxy-glucose positron emission tomography and computer tomography (FDG-PET/CT) examinations; (4) patients included in the final analysis were those who underwent surgery after completion of PST.

Clinical TNM was assessed by using routinely applied diagnostic imaging modalities (X-ray and ultrasound mammography, and FDG-PET/CT).

The study was ethically approved by the Semmelweis University Institutional Review Board (No.: SE TUKEB 120/2013).

### Histopathological Analysis and Pathological Response Evaluation

Before PST detailed histological characterization was performed on the core biopsy samples (i.e. histological type, nuclear grade, tubule formation score, mitotic index, presence or absence of in situ carcinoma component, perineural and lymphovascular invasion). Additionally, the core-biopsy samples were evaluated for the presence of tumor infiltrating lymphocytes (TIL) in the stroma of the tumors according to the TILs Working Group guideline published in 2015 [27].

In surgical samples, if residual tumor was present, histological characterization was repeated; in addition, residual tumor size and nodal stage were also assessed with the evaluation of tumor-free margins. For pathological response evaluation the surgical samples were analyzed according to national consensus recommendations [28] based on the Pinder [29] response classification: the response of the primary tumors (TR1–3 categories) and regional lymph nodes (NR1–4 categories) were assessed separately. pCR was diagnosed only if no viable invasive tumor cells were identified in the breast specimen after the whole tumor bed and the resected axillary lymph nodes were embedded and thoroughly investigated. Presence of ductal carcinoma in situ (DCIS) was allowed, the pCR was defined as ypT0/Tis [30].

### Immunohistochemistry

Formalin-fixed, paraffin-embedded pre-treatment core biopsy samples were examined. Immunohistochemistry (IHC) was routinely performed to evaluate hormone receptor – estrogen (ER) and progesterone (PR) – status, as well as HER2 expression according to international guidelines. Hormone receptor positivity was confirmed if Allred score was above or equal to 3 [31]. HER2 overexpression was defined as IHC 3+. HER2 1+ or 0 tumors were considered Her2 negative. For IHC 2+ samples, fluorescent in situ hybridization (FISH) was performed to confirm gene amplification. HER2 status was defined according to the ASCO/CAP Guideline valid at the time of diagnosis, i.e. HER2-positive patients treated between January 2008 and November 2013 were identified according to the 2007 ASCO/CAP Guideline [32] and from then on according to the Guideline published in October 2013 [33]. Using these parameters – and the value of the Ki-67 LI, described below – biological subtype of the tumors was defined according to the recommendations of the 13th St. Gallen International Breast Cancer Conference [2].

Ki-67, MCM2, Cyclin A and PHH3 were stained as described previously by Tőkés et al. [21]. Proliferation markers were scored by two investigators (TT and AMT) as described earlier [34, 35]. Briefly, every slide was assessed visually and the proportion of positive cells was determined by counting approximately 500 tumor cells at 400x magnifications. For Ki-67, MCM2 and Cyclin A markers, a cell was considered positive if any nuclear signal was observed, similar to the studies of Ali et al. [36] and Tőkés et al. [9]. Intensity was scored based on the recommendation of Dowsett et al. [34]. Regarding PHH3 – over quantifying the proportion of positive cells as described above – an additional evaluation of mitotic count was also performed in 10 high-powered fields (HPF) at 400x magnifications, as described earlier by Bossard et al. [15]. Briefly, only those nuclei were counted as PHH3 positive which were characterized by strong and dense staining of chromatin clumps, representing prophase, metaphase, anaphase and telophase. PHH3 nuclei with fine granular staining were excluded from the counting as representing interphase nuclei.

### Statistical Analysis

All applied statistical tests were two-sided and *p* values <0.05 were considered significant. Data were expressed as mean ± standard deviation (SD). Normality was tested by using Shapiro-Wilks test. Connections between clinicopathological characteristics and expressions of the investigated cell cycle proteins were assessed by using Mann-Whitney and Kruskal-Wallis tests. To compare pCR and non-pCR patient groups Mann-Whitney tests were performed.

PFS was evaluated after 2 years follow-up and at the end of the follow-up period (31 May 2017), together with OS. The data of those patients who did not experience progression or cancer-related death was censored at the last control visit at the Oncology Center of Semmelweis University. Patients who experienced progression and those who did not were compared by means of clinicopathological characteristics using Mann-Whitney and Kruskal-Wallis tests. Moreover logistic regression was performed to assess the prognostic potential of the tested cell-cycle markers to PFS. For survival analyses, Kaplan-Meier product limit methods were used with log-rank tests to compare patient groups defined by the high or low expression of proliferation markers. For Ki-67 we used cut-off points described earlier by our research group (for Ki-67 LI 20% for PFS and 30% for OS [23]); for the other markers we applied earlier published prognostic cut-off points as follows: for MCM2 30% [7], for Cyclin A 10.5% [26], for PHH3 13 mitosis/10 NNL [17].

Additionally, based on the hypothesis of Loddo et al. [7] we grouped our patients in four clusters concerning the expression of the investigated proliferation markers over the routinely used Ki-67 LI. For subgrouping we also used the above described prognostic cut-off points, as follows:

- Group I) low MCM2 expression with any Cyclin A, any PHH3 expression
- Group II) high MCM2 but low Cyclin A and low PHH3 expression
- Group III) high MCM2 with high Cyclin A and low PHH3 expression
- Group IV) high MCM2, high Cyclin A and high PHH3 expression
- Group V) other tumors (with high MCM 2, low Cyclin A and high PHH3 expression).

Subgroups were compared using heatmap analysis, regarding the distribution between biological subgroups and clinical outcome.

Microsoft Excel 2010 (Microsoft Corp., Redmond Washington, USA) and Statistica 13.2 (StatSoft Inc., Tulsa, Oklahoma, USA) software were used for data collection and processing.

## Results

### Clinicopathological Characteristics and Expression of the Cell-Cycle Markers

A total of 52 breast cancer patients (mean age: 48.02 ± 10.51 years) were enrolled in our current study (Table 1). Dominantly grade 3 (32/52, 62%) invasive breast carcinomas of no special type (IBC NST) (48/52, 92%) were evaluated. 33 tumors were ER-positive, 30 PR-positive. HER2 positivity was identified in 21 cases. Seven tumors were luminal A-like and 27 were luminal B-like – from these 12 were luminal B-proliferative and 15 were luminal B-HER2 positive. 6 tumors were classified as HER2-positive subtype and 12 were triple negative.

**Table 1:** Clinicopathological characteristics of the tumors (*n* = 52)

Characteristics	No.	%
Clinical T categories		
T1c	5	9.6
T2	39	75
T3	5	9.6
T4	3	5.8
Clinical N categories		
N0	21	40.4
N1	19	36.5
N2	5	9.6
N3	7	13.5
Histology		
Invasive breast carcinomas of no special type (IBC NST)	48	92.3
Other	4	7.7
Grade		
1	0	0
2	20	38.5
3	32	61.5
Biological subtype		
Luminal A	7	13.5
Luminal B/proliferative	12	23.1
Luminal B/Her2-positive	15	28.8
Her2-positive	6	11.5
Triple negative	12	23.1
Primary systemic therapy		
Taxane-anthracycline combination	19	36.5
Taxane-platinum combination	15	28.8
Trastuzumab-based combination	11	21.2
Other	7	13.5
Surgical therapy		
Mastectomy	35	67.3
Breast conserving surgery	17	32.7
Axillary block dissection	43	82.7
Sentinel lymph node biopsy	9	17.3

By analyzing the relationship between the expression of the investigated cell-cycle proteins and the known predictive and prognostic factors (i.e. cT and cN categories; histological type; nuclear grade; ER, PR and HER2 status; biological subtype) in pre-therapy core-biopsies (Table 2) we found significantly different expression of MCM2 and PHH3 according to cT categories: smaller lesions showed more pronounced tumor proliferation. Initial clinical N stage did not show any correlation with proliferation activity. Every cell cycle marker was significantly higher in grade 3 tumors compared to grade 2 lesions. Regarding hormone receptor status, we found that all markers but PHH3 showed higher expression in ER and PR negative tumors than in hormone receptor positive cancers. HER2 status did not show any correlation with tumor proliferation. Triple negative carcinomas showed higher proliferation activity than any other subtypes with every investigated marker.

**Table 2:** Relationship between the clinicopathological characteristics and the expression of cell-cycle markers in core biopsy specimens

Feature (No. of tumors)	Ki-67 mean	MCM2 mean	Cyclin A mean	PHH3 mean
Clinical T categories				
T1 (5)	70.00	70.00	43.00	16.00
T2 (39)	48.52	55.38	24.59	8.38
T3 (5)	46.00	50.00	20.00	6.00
T4 (12)	31.66	16.67	16.67	8.34
p value	**0.2157**	**0.0225**	**0.0830**	**0.0468**
Clinical N categories				
N0 (21)	42.86	49.29	21.52	8.43
N1–3 (31)	53.77	57.26	28.13	9.19
p value	**0.1366**	**0.2578**	**0.2135**	**0.3739**
Histology *				
IDC (48)	50.98	55.00	26.39	9.17
other (4)	30.00	42.50	14.25	5.50
Nuclear grade				
II (20)	32.60	38.35	17.20	6.85
III (32)	59.84	63.91	30.63	10.63
*p* value	**<0.0001**	**<0.0001**	**0.0003**	**0.0424**
ER status				
positive (33)	37.33	45.61	19.97	7.64
negative (19)	70.26	68.68	35.00	11.05
p value	**<0.0001**	**0.0003**	**0.0011**	**0.0544**
PR status				
positive (30)	36.90	46.83	19.47	7.83
negative (22)	66.36	66.86	33.64	10.32
p value	**<0.0001**	**0.0089**	**0.0013**	**0.1691**
Her2 status				
positive (21)	47.86	50.00	24.52	8.67
negative (31)	50.39	56.77	26.09	9.03
p value	**0.8245**	**0.2500**	**0.9558**	**0.7958**
Triple negativity				
no (40)	41.30	47.63	12.72	7.55
yes (12)	76.25	75.42	37.92	13.34
p value	**<0.0001**	**<0.0001**	**0.0046**	**0.0053**
Biological subtype				
Luminal A (7)	19.57	37.14	8.43	4.71
Lum.B-prolif. (12)	42.50	49.58	24.58	7.25
Lum.B-Her2 + (15)	41.66	48.00	21.00	9.13
Her2-positive (6)	63.33	55.00	33.33	7.50
Triple negative(12)	76.25	75.42	37.92	13.34
p value	**<0.0001**	**0.0026**	**0.0004**	**0.0119**
Tumor infiltrating lymphocytes (TIL)^#^				
TIL ≤ 1% (18)	38.33	46.67	21.61	7.61
TIL 1–20% (19)	57.37	53.95	28.42	10.37
TIL >20% (6)	60.00	58.33	34.17	7.17
p value	**0.0402**	**0.9676**	**0.4744**	**0.9970**

Underlined: statistically significant difference

*due to low case numbers and statistical power we did not perform a statistical comparison

# missing in 9 patients

We also analyzed the correlations between the presence of stromal TILs and the cell cycle activity of the primary tumors. Tumors with increased TIL score showed significantly higher Ki-67 expression (*p* = 0.04). The other three cell-cycle markers were also higher in tumors with high TIL score than those with lower scores, but this tendency was not significant (Table 2). Core-biopsy TIL was significantly higher in those patients who achieved pCR compared to those with residual tumors (13.52% vs. 2.46%, respectively; *p* = 0.0008).

### Pathological Response Rates and Cell Cycle Marker Expression

All patients were treated with PST, most commonly in 3-week schedules, for 6 or 8 cycles. Mostly taxane-based regimens were administered (*n* = 48). After completion of PST, every included patient gave consent to surgery. Regarding the final histological analyses, 17 patients achieved pCR (17/52; 32.7%). We detected residual tumors in 35 cases (35/52, 67.3%), from these in 4 cases (7%) only the regional lymph nodes contained malignant cells. In 33 patients we did not detect any lymph node metastasis after the PST (ypN0 = 33/52; 63.5%), in 19 patients nodal involvement was confirmed, but from these in one case only a micrometastasis was detected. Among residual tumors, dominant subtypes were the luminal ones: 22/35 (63%) tumors were luminal B-like – from these 11 were luminal B-proliferative and 11 were luminal B-HER2 positive – and 7/35 (20%) lesions were luminal A-like.

We found significantly higher initial Ki-67, MCM2, Cyclin A and PHH3 expression in the pCR cases compared to non-pCR patients (*p* < 0.0001, *p* = 0.0024, *p* = 0.0032 and *p* = 0.0294, respectively). Additionally, strong significance was found when the initial cell cycle activity (Ki-67, MCM2, Cyclin A and PHH3 expression) of ypT0/is patients was compared with other ypT stages. However, we did not find any significant correlation between the initial cell-cycle activity and the lymph node involvement (ypN0 vs. other ypN stages) after the PST **(**Table 3**)**.

**Table 3:** Relationship between the rate of tumor response and the expression of cell cycle markers in core biopsy specimens

Feature (No. of tumors)	Ki-67 mean	MCM2 mean	Cyclin A mean	PHH3 mean
pCR (*n* = 17)	68.53	67.65	35.00	11.06
non-pCR (*n* = 35)	40.06	47.43	20.83	7.830
*p value (pCR* vs *non-pCR)*	**<0.0001**	**0.0024**	**0.0032**	**0.0294**
ypT categories^1^				
ypT0/is (*n* = 21)	65.95	67.38	34.52	10.86
ypT1 (*n* = 11)	47.27	49.09	23.64	9.73
ypT2 (*n* = 13)	34.00	47.31	18.23	5.38
ypT3 (n = 4)	31.25	37.50	9.25	5.50
ypT4 (n = 2)	35.00	25.00	20.00	7.50
p value (all compared)	**0.0031**	**0.0094**	**0.0017**	**0.0169**
p value (T0/is vs T+)*	**0.0001**	**0.0005**	**0.0003**	**0.0056**
Tumor response of the primary breast lesions				
TR1a and 1b (*n* = 21)	65.95	67.38	34.52	10.86
TR2a (n = 5)	31.00	58.00	22.00	12.00
TR2b (*n* = 7)	46.43	43.57	22.86	7.43
TR2c (n = 11)	43.64	43.18	18.82	6.91
TR3 (*n* = 8)	27.75	40.63	15.25	5.75
p value (all compared)	**0.0015**	**0.0091**	**0.0065**	**0.0291**
p value (TR2a-3 compared)	***0.3500***	***0.5739***	***0.5411***	***0.3128***
ypN categories^2^				
ypN0 (*n* = 33)	54.24	58.64	28.24	9.55
ypN1 (*n* = 10)	40.7	50.50	18.70	7.70
ypN2 (*n* = 6)	40.83	35.00	21.67	8.33
ypN3 (n = 2)	32.50	55.00	22.50	5.00
p value (all compared)	***0.3275***	***0.1753***	***0.3149***	***0.7039***
p value (N0 vs N+)#	***0.0743***	***0.0810***	***0.0846***	***0.5912***
Remission rate in the axillary lymph nodes				
NR1 (n = 33)	54.24	58.64	28.24	9.55
NR3 (n = 9)	47.78	45.56	23.00	8.00
NR4 (n = 10)	34.7	46.5	18.50	7.50
p value (all compared)	***0.1162***	***0.1929***	***0.1990***	***0.8292***
p value (NR3 vs NR4)	***0.1564***	***0.8421***	***0.4469***	***0.6607***

Underlined: statistically significant difference

[1] one specimen with a micrometastasis was excluded from these analyses

[2] one specimen with a micrometastasis was excluded from these analyses

*ypT0/is patient group compared to patients with pathological stages ypT1a or greater

# ypN0 patient group compared to patients with pathological stages ypN1a or greater

We also assessed residual tumors according to the degree of tumor response. We assessed the residual primary tumors and regional lymph nodes separately. From the 31 cases with residual primary tumors we detected a therapeutic response to PST in 23 patients (23/31, 74%) and only 8 patients showed no evidence of tumor response (TR3: 8/31, 26%). The partial responder tumors were distributed between TR2a, TR2b and TR2c response categories unevenly (5, 7 and 11 patients, respectively). We did not find significant correlation between the degree of tumor response detected in the primary lesions after surgery and the initial cell-cycle activity in the core-biopsies. Amongst the cases with node positive disease detected after PST, only nine patients showed signs of therapeutic response (9/19, 47%) while in ten patients (10/19, 53%) the nodal metastases showed no evidence of response to therapy. Regarding regional lymph nodes, we did not find any correlation between the NR response status and the initial cell-cycle activity, similarly to the primary lesions. (Detailed results are presented in Table 3).

### Survival Analysis

Median follow-up time was 62 months. We detected disease progression in 12 cases (12/52; 23%). From these, 8 patients belonged to the non-pCR patient group after PST. However, we did not find any significant differences between these 12 patients and the rest of the patients (40/52) regarding the initial expression of Ki-67 (*p* = 0.277), MCM2 (*p* = 0.583), Cyclin A (*p* = 0.724) or PHH3 (*p* = 0.267) measured in the core-biopsy samples. Additionally, from the 12 cases with progression 7 (7/12; 58%) were considered as early onset relapse (PFS ≤ 2 years). From these seven cases in 6 patients the expression of every tested cell cycle markers was high in the core-biopsy samples, but we could not prove any significant predictive potential of the initial Ki-67 (*p* = 0.097), MCM2 (*p* = 0.172), Cyclin A (*p* = 0.932) or PHH3 (*p* = 0.380) expression towards early relapse.

Five patient deceased during the follow-up (9.6%), all death were cancer-related and all of these patients experienced non-pCR after the PST. In four of these five patients (80%), every cell cycle marker was elevated in their pre-treatment core-biopsy samples.

Based on Kaplan-Meier analysis and log rank tests we could not prove any significant prognostic potential of the tested cell cycle markers towards PFS or OS (see **Additional file 1**).

### Pattern Analysis

We performed further subgrouping of the patients based on the expression pattern of the investigated – relatively phase specific – cell-cycle proteins. The subgroups and distribution of the patients amongst the subgroups were as follows:

- Group I) low MCM2 expression with any Cyclin A, any PHH3 expression: *n* = 9
- Group II) high MCM2 but low Cyclin A and low PHH3 expression: *n* = 2
- Group III) high MCM2 with high Cyclin A and low PHH3 expression: n = 2
- Group IV) high MCM2, high Cyclin A and high PHH3 expression: *n* = 34
- Group V) other tumors (with high MCM 2 with low Cyclin A and high PHH3 expression): *n* = 5

**Fig. 1:**
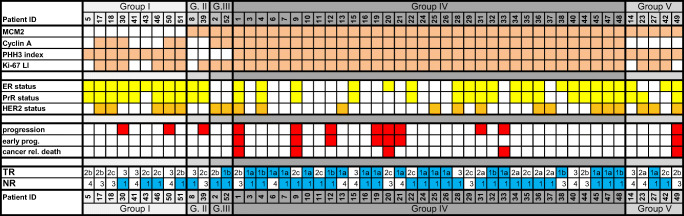
**Heat map**. In the first block the peach colored cells represent those tumors showing high MCM2, Cyclin A, PHH3 and Ki-67 expression. In the second part yellow squares represent the ER, PR positive tumors and orange ones mark the HER2 positive lesions. In the third part red squares represent progressed cases. In the last part tumor response is detailed – in the primaries (TR) and in the lymph node region (NR) according to the Pinder classification

A heatmap was designed to compare the four patient groups regarding biological tumor subtypes and clinical outcome (Fig. 1). Hormone receptor positive tumors with favorable outcome were more frequent in Group I and II. In comparison, only Group IV contained triple negative tumors and this patient group showed the most favorable response to PST – almost all pCR cases grouped together into this most actively proliferating patient subgroup.

Nonetheless, regarding the rate of progression the most unfavorable results were also experienced in Group IV; from 34 patients 9 showed progression (26%), whilst in the other three patient groups (Group I, II, III and V) only two, one, zero and one patient showed a relapse, respectively.

**Fig. 2:**
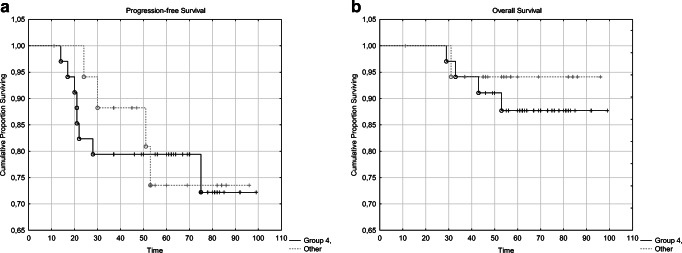
Comparison of the PFS (**a**) and OS (**b**) of patients in Group IV (containing patients with high MCM2, high Cyclin A and high PHH3 expression) vs. the other investigated patient groups (Group I, II, III and V)

Kaplan-Meier plots comparing the PSF and OS of the patients in Group IV with the others are shown on Fig. 2. We did not find any significant differences between the survival of these two patient groups (*p* = 0.907 and *p* = 0.551, respectively).

## Discussion

Relationship between the clinicopathological characteristics of breast tumors and the routinely used Ki-67 proliferation marker has already been established. However, due to the known analytical limitations of Ki-67 LI [5, 6], several other proliferation markers were already tested, but only a few studies analyzed the relationship of these cell-cycle markers and the known predictive and prognostic factors of locally advanced breast cancer.

In our study, while analyzing core-biopsy samples of locally advanced breast cancers, we found that smaller lesions (cT1 and cT2 tumors) showed higher proliferation activity measured by all investigated markers than larger tumors. In case of MCM2 and PHH3 these correlations were significant. If the axillary lymph node status was >cN0, as per clinical-imaging investigations, the expression of every proliferation marker was higher compared to clinically node-negative (cN0) cases, but the relationship was not significant. Earlier published results are contradictory regarding the investigated markers of our study. Wiesner et al. analyzed the data of more than one thousand patients and found significant correlation between the expression of Ki-67 and tumor size as well as axillary involvement [37]. Other studies only described a relationship with tumor size but not with cN categories [38]. Correlation between the expression of MCM2 and tumor size was already described [39], like in case of PHH3 [17], but their connection with axillary involvement was not proved. Regarding Cyclin A neither tumor size nor axillary involvement was proved to be connected with the protein expression in one of the published studies [26], but two other studies proved a positive connection with the cT categories [25, 40].

The expression of all examined cell-cycle markers was higher in high-grade tumors, in agreement with earlier results [17, 26, 37, 39, 41]. It has to be highlighted that in case of grade 2 cancers with uncertain biological aggressiveness Ki-67 LI can be used for further subgrouping into different prognostic groups [37]. Other markers applied in our study can also be applicable for this clinical purpose, therefore the selection of suitable patients for PST could be more accurate.

In case of hormone receptor negative (ER and PR negative) tumors every proliferation marker showed significantly higher expression – except PHH3 – compared to hormone receptor positive carcinomas. This correlation has already been described in case of Ki-67 [5, 37, 38], MCM2 [39], Cyclin A [25, 26, 40] and even of PHH3 [17, 39].

In our study we did not find a significant correlation between HER2 positivity and the expression of the investigated markers. Earlier studies also resulted in contradictory findings regarding the relationship between Ki-67 LI and HER2 expression. In some studies a positive correlation was described between these markers, while others did not find any correlation [36, 37]. Regarding MCM2 and Cyclin A similar contradictory results were published [25, 26, 36, 39]. PHH3 expression did not show any correlation with HER2 positivity [36], but it has been desribed that PHH3 expression positively correlates with nuclear atypia and negatively with tubule forming ability [17, 39].

Regarding biological subtypes defined in the core-biopsy samples we detected significantly higher expressions of all four cell-cycle markers in triple negative tumors, while in case of luminal A-like subtypes we found the lowest proliferation activity measured by every tested markers. We did not find any earlier published study on MEDLINE directly investigating the correlations between biological subtypes and MCM2, Cyclin A or PHH3 expression. Regarding the expression of Ki-67 LI it is already descibed that luminal A-like tumors (with the most favourable prognosis) have usually got low Ki-67 LI by definition. [2, 42] Notably, in these tumors pCR is rare when PST is applied [2, 43]. High Ki-67 LI can also be applied to define a patient subgroup amongst high grade, triple negative invasive breast carcinomas with high pCR rate but worse prognosis [44, 45]. The high expression of Ki-67, MCM2, Cyclin A and PHH3 is also in correlation with high grade and triple negativity, therefore all tested marker seem to be potentially applicable to delineate a patient group with biologically aggressive breast tumors to be suitable to PST.

TIL ratio of breast malignancies has been frequently investigated lately due to its possible connection to the efficiency of immunotherapies and its strong prognostic potential for favourable clinical outcome – but this prognostic potential depends on the biological subtypes of the tumors. Lymphocyte predominant tumors have favorable clinical response to chemotherapy amongst triple negative cancers and to trastuzumab in HER2 positive cases, but not in hormone receptor positive breast malignancies [27, 46]. In our study we found significantly higher infiltration of TIL in the core biopsies of tumors that achieved pCR. When assessing the relationship between the presence of TIL in the primaries and proliferation activity of these tumors, we did not find any earlier published study on MEDLINE directly investigating the correlations between TIL ratio and MCM2, Cyclin A or PHH3 expression. Regarding Ki-67, it has been recently proved that the presence of immune cells are related to the high proliferation activity of breast tumors – together with high grade and hormone receptor negativity [47]. Our study confirmed this correlation, but did not find any significant relationship between the presence of TIL and the expression of MCM2, Cyclin A or PHH3.

Regarding the predictive and prognostic potential of the investigated markers our study partially supported, but partially disproved earlier published results. As mentioned earlier initial Ki-67 LI have already been proved to be predictive for pCR in breast cancer patients [18–20]. In our study pCR rate was relatively high (17/52, 32.7%) in the surgical specimens compared to international results [1–3]. HER2 positive and triple negative tumors were predominant in the pCR patient group and amongst non-pCR patients the majority had breast cancer with luminal characteristics– in accordance with the international literature [1–3]. In our current as well as in our earlier published results [21] we proved that besides Ki-67, the higher expression of MCM2, Cyclin A and PHH3 also accurately predicts the pCR after PST.

However, we did not find any significant correlation between the degree of the tumor response in the non-pCR patients (neither in the primary tumors nor the involved axillary lymph nodes) and the initial cell-cycle activity of the tumors. Besides the strong predictive potential of high cell-proliferation activity towards pCR, we revealed a lack of any reliable biomaker to differentiate between tumors with partial tumor remission. In the international literature we did not find any earlier published study directly investigating the correlations between TR and NR response grading and MCM2, Cyclin A or PHH3 expression. Regarding the Ki-67 LI, in the earlier study of our research group we could not find any correlation between Ki-67 LI and rate of tumor response. However, the difference was significant when the pCR group and partial pathological responder group were compared, with the exclusion of those tumors which did not show any reactions to the applied neoadjuvant regimens, in agreement with the results of Balmativola et al. [20, 23].

Concerning prognostic potential it is well-known that high expression of Ki-67 is associated with poor prognosis and earlier onset of metastatic progression [18, 19]. However, contradictory data are presented about the reliability of Ki-67 LI to assess tumor proliferation: Ki-67 LI has a relatively low inter-laboratory reproducibility and there is an active debate about the predictive and prognostic cut-off values advised for the daily routine [5, 6]. Based on our study it can be stated that progression was more frequent amongst the tumors with higher proliferation activity, but the association was not significant.

Nonetheless, Loddo et al. [7] already suggested a novel approach in the assessment of core-biopsy samples using proliferation activity to differentiate between patient groups with different therapeutic sensitivity and prognosis. They assessed different, relatively phase-specific markers of the cell-cycle paralelly and form subgroups of the patients based on the relative ratio of tumor cells overexpressing G1/S/G2 and M phase markers. Based on their hypothesis we formed five patient groups concerning the expression of MCM2, Cyclin A and PHH3. Our results suggested that cell cycle marker-based subgrouping performed on core-biopsy samples could be used to accurately differentiate between tumors with different response to PST and prognosis. Additionally, we have to highlight that in our study we mostly investigated tumors fit to the ‘phenotype III/actively cycling’ tumor group of the Loddo study [7] (this group corresponds to Group III, IV an d V of our study) – being in harmony to the desirable goal that every patient of our study was selected to be an ideal candidate of PST. Only two patients could be classified to ‘phenotye II/G1-delayed/arrested’ tumors of the Loddo study [7] (this group corresponds to Group II of our study) in whom classic S and M cell-cycle-phase-targeted agents − like the widely used taxane and anthracyclin chemotherapies − may not be as effective than in actively cycling cells (amongst these patients pCR was not observed in our study and the response to the applied therapy was minimal). These tumors are more likely to benefit from G1-phase targeted agents or non-cell-cycle-specific anticancer drugs [7].

The limitation of our study is the few disease related events during the follow-up period of our patients, therefore the statistical power of the performed survival analyses is weak. Additionally, to further investigate the clinical usefulness of the above described cell-cycle based subgrouping further studies are needed with higher number of included patients.

## Conclusions

Selecting patients diagnosed with breast cancer for PST is a critical problem in the daily practice. In our current study the pCR rate was relatively high, compared to international data; however, further assessment of the pretreatment core-biopsies is still advised to define supplementary biomarkers to increase the efficiency of the patient selection.

In our current study, we evaluated nuclear protein markers either expressed throughout (Ki67, MCM2), from post G1 (Cyclin A) or in M-phase (PHH3) of the cell cycle and their connections with the routinely used predictive and prognostic factors in breast cancer. Significant associations were described between the cell-cycle activity and the cT stage, grade, hormone receptor status and triple negativity of the investigated tumors. Novel biomarkers of tumor response and survival such as the presence of TILs – which was associated with more frequent occurrence of pCR – are also related to cell-proliferation, however, only Ki-67 showed significant correlation with stromal TIL.

We already proved that Ki-67, MCM2, Cyclin A and PHH3 are good predictors of pCR after the PST, however, not only pCR, but also rate of partial tumor remission is prognostic for the clinical outcome. In our current study, we did not find significant correlation between the degree of tumor response and the initial cell-cycle activity.

Regarding prognostic significance, the pattern of cell-cycle protein expression could be promising as predictive and prognostic tool to be applied before the initiation of PST as well as be useful to choose the therapeutic agent applied in this setting.

## Electronic supplementary material

ESM 1 (DOCX 118 kb)

## Data Availability

The datasets generated during and/or analysed during the current study are available from the corresponding author on reasonable request.

## Abbreviations

ASCO/CAP: American Society of Clinical Oncology/College of American Pathologists
DCIS: ductal carcinoma in situ
ER: estrogen receptor
FDG-PET/CT: ^18^F-fluorodeoxy-glucose positron emission tomography and computer tomography
FISH: fluorescent in situ hybridization
HPF: high-powered fields
IBC NST: invasive breast carcinomas of no special type
IHC: immunohistochemistry
Ki-67 LI: Ki-67 labeling index
MCM2: minichromosome maintenance protein
OS: overall survival
pCR: pathological complete remission
PHH3: phosphohistone-H3
PR: progesterone receptor
PFS: progression-free survival
PST: primary systemic therapy
ROC: Receiver Operating Characteristic
SD: standard deviation
TIL: tumor infiltrating lymphocytes
