# Supplementary material for: Prognostic and Clinicopathological Correlations of Cell Cycle Marker Expressions before and after the Primary Systemic Therapy of Breast Cancer
Source: Pathol Oncol Res. 2019 Aug 24;26(3):1499–510. doi: 10.1007/s12253-019-00726-w (PMC7297700; doi:10.1007/s12253-019-00726-w)
Supplement: Supplementary file 1 — (DOCX 118 kb) [file 12253_2019_726_MOESM1_ESM.docx]

**SUPPLEMENTARY FIGURES**

**For the paper titled: *Prognostic and clinicopathological correlations of cell cycle marker expressions before and after the primary systemic therapy of breast cancer***

**AUTHORS:**

Tímea Tőkés [1], Anna-Mária Tőkés [2], Gyöngyvér Szentmártoni [1], Gergő Kiszner [3], Béla Ákos Molnár [4] Janina Kulka [2], Tibor Krenács [3], Magdolna Dank [1]

[1] Semmelweis University, Oncology Center; Tömő utca 25-29, Budapest, Hungary, H-1083.

[2] Semmelweis University 2nd Department of Pathology; Üllői út 93, Budapest, H-1091.

[3] Semmelweis University 1st Department of Pathology and Experimental Cancer Research; Üllői út 26, Budapest, H-1085

[4] Semmelweis University 1st Department of Surgery, Üllői út 78/A, Budapest, H-1083

***Corresponding author**

Tímea Tőkés MD, PhD; Semmelweis University Oncology Center

Address: Tömő utca 25-29. 4^th^ floor, H-1083, Budapest, Hungary.

E-mail: timi.tokes@gmail.com , tokes.timea@med.semmelweis-univ.hu;

Phone: +36206663567; Fax: +36208251730.

***Supplementary FIGURE 1****.*

*Progression-free survival differences between the patient groups defined by the expression of Ki-67, MCM2, Cyclin A and the values of the PHH3 index in the core biopsy samples*


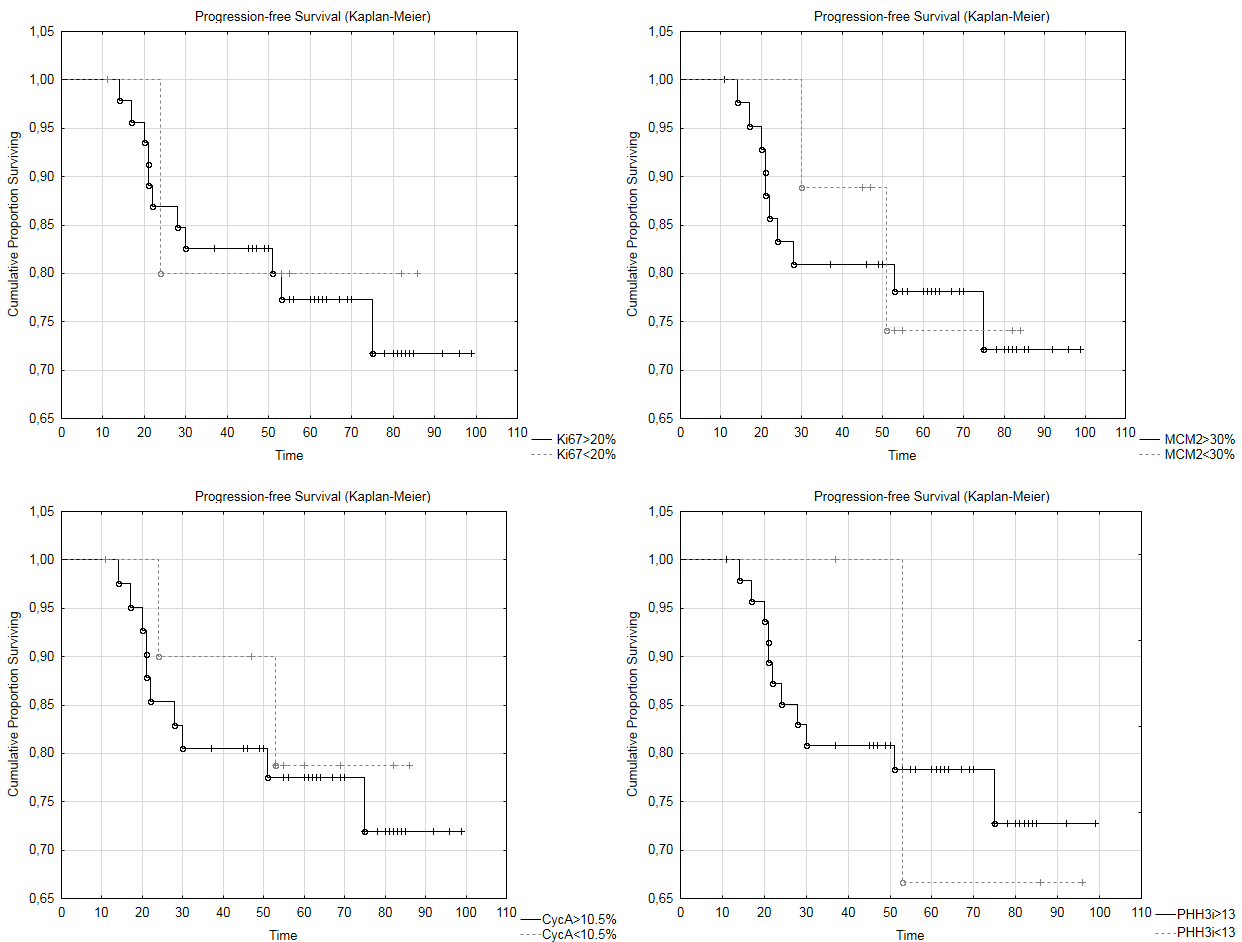


***Supplementary FIGURE 2.***

*Overall survival differences between the patient groups defined by the expression of Ki-67, MCM2, Cyclin A and the values of the PHH3 index in the core biopsy samples*

*
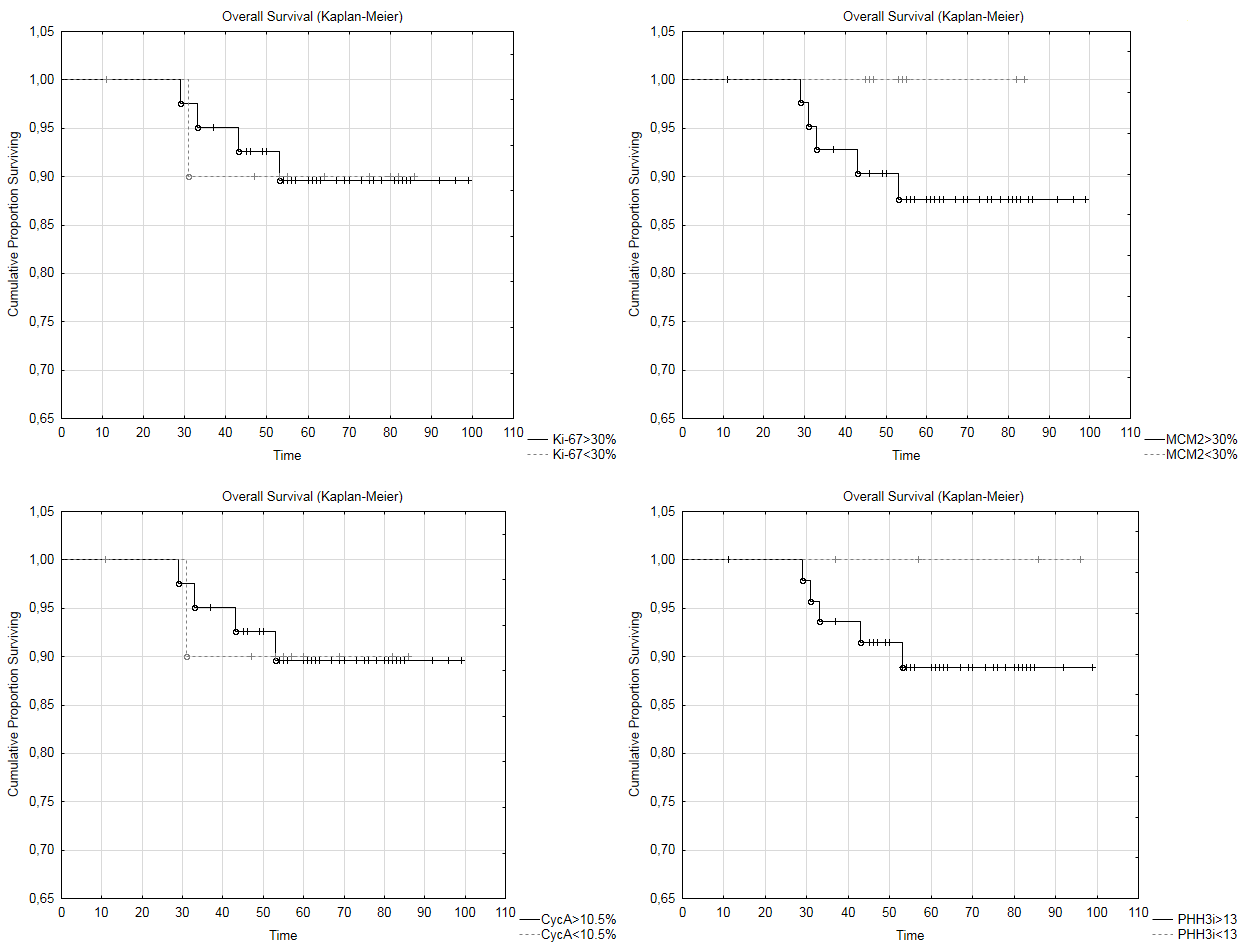
*
